# Artificial Intelligence in Ophthalmology: A Meta-Analysis of Deep Learning Models for Retinal Vessels Segmentation

**DOI:** 10.3390/jcm9041018

**Published:** 2020-04-03

**Authors:** Md. Mohaimenul Islam, Tahmina Nasrin Poly, Bruno Andreas Walther, Hsuan Chia Yang, Yu-Chuan (Jack) Li

**Affiliations:** 1 Graduate Institute of Biomedical Informatics, College of Medical Science and Technology, Taipei Medical University, Taipei 110, Taiwan; d610106004@tmu.edu.tw (M.M.I.); d610108004@tmu.edu.tw (T.N.P.); itpharmacist@gmail.com (H.C.Y.); 2 International Center for Health Information Technology (ICHIT), Taipei Medical University, Taipei 110, Taiwan; 3 Research Center of Big Data and Meta-Analysis, Wan Fang Hospital, Taipei Medical University, Taipei 110, Taiwan; 4 Department of Biological Sciences, National Sun Yat-Sen University, Gushan District, Kaohsiung City 804, Taiwan; bawalther2009@gmail.com; 5 Department of Dermatology, Wan Fang Hospital, Taipei 110, Taiwan; 6 TMU Research Center of Cancer Translational Medicine, Taipei Medical University, Taipei 110, Taiwan

**Keywords:** retinal vessel, deep learning, diabetes mellitus, convolutional neural network, artificial intelligence

## Abstract

Background and Objective: Accurate retinal vessel segmentation is often considered to be a reliable biomarker of diagnosis and screening of various diseases, including cardiovascular diseases, diabetic, and ophthalmologic diseases. Recently, deep learning (DL) algorithms have demonstrated high performance in segmenting retinal images that may enable fast and lifesaving diagnoses. To our knowledge, there is no systematic review of the current work in this research area. Therefore, we performed a systematic review with a meta-analysis of relevant studies to quantify the performance of the DL algorithms in retinal vessel segmentation. Methods: A systematic search on EMBASE, PubMed, Google Scholar, Scopus, and Web of Science was conducted for studies that were published between 1 January 2000 and 15 January 2020. We followed the Preferred Reporting Items for Systematic Reviews and Meta-analyses (PRISMA) procedure. The DL-based study design was mandatory for a study’s inclusion. Two authors independently screened all titles and abstracts against predefined inclusion and exclusion criteria. We used the Quality Assessment of Diagnostic Accuracy Studies (QUADAS-2) tool for assessing the risk of bias and applicability. Results: Thirty-one studies were included in the systematic review; however, only 23 studies met the inclusion criteria for the meta-analysis. DL showed high performance for four publicly available databases, achieving an average area under the ROC of 0.96, 0.97, 0.96, and 0.94 on the DRIVE, STARE, CHASE_DB1, and HRF databases, respectively. The pooled sensitivity for the DRIVE, STARE, CHASE_DB1, and HRF databases was 0.77, 0.79, 0.78, and 0.81, respectively. Moreover, the pooled specificity of the DRIVE, STARE, CHASE_DB1, and HRF databases was 0.97, 0.97, 0.97, and 0.92, respectively. Conclusion: The findings of our study showed the DL algorithms had high sensitivity and specificity for segmenting the retinal vessels from digital fundus images. The future role of DL algorithms in retinal vessel segmentation is promising, especially for those countries with limited access to healthcare. More compressive studies and global efforts are mandatory for evaluating the cost-effectiveness of DL-based tools for retinal disease screening worldwide.

## 1. Introduction

### 1.1. Rationale

Visual impairment is a public health concern that has a negative impact on physical and mental health [1]. Visual impairment is associated with a high risk of chronic health conditions, including death. The prevalence and economic burden of visual impairment are exponentially increasing with an increasing number of aging populations [2]. It is estimated that the number of people with visual impairment will double by 2050 [3]. Several potential factors, such as cataract, age-related macular degeneration (AMD), diabetic retinopathy (DR), and glaucoma, are responsible for an increased risk of blindness [4,5]. This highlights the important public health burden that visual impairment and blindness place on our health care system. Therefore, the early detection and quantitative diagnosis of retinal diseases can help to develop more preventive measures, thereby reducing the number of newly diagnosed cases and the associated financial burden [6].

### 1.2. Solution Statement

Retinal fundus images are often used for early diagnosis of different ophthalmologic diseases, including DR and glaucoma [7]. Among various features in digital fundus images, retinal blood vessels provide useful information that is an important prerequisite for a number of clinical applications [8]. However, manual segmentation of retinal vessels by a trained human expert is time-consuming and highly variable [9,10]. The lack of human observers, infrastructure, and awareness are key challenges that need to be overcome. Over the past decades, automatic retinal vessel segmentation methods were mainly classified into two categories: supervised and unsupervised. Unsupervised methods are always dependent on threshold filter responses, handcrafted features, or other rule-based techniques. In contrast, supervised methods train a classifier to obtain discrimination between the vessel and non-vessel pixels. Recently, deep learning (DL) has achieved tremendous diagnostic performance in segmenting the retinal vessel [11,12]. The diagnostic accuracy of DL in retinal vessel segmentation has been shown to be comparable to the accuracy that was achieved by human experts. DL-based automatic systems offer potential benefits by reducing the manual work and achieving faster segmentation with reduced costs and resources. DL-based automatic tools can be incorporated into real-world screening programs that are not widely implemented or routinely practiced [13].

### 1.3. Goal of Investigation

Herein, we report the results of a comprehensive systematic review of DL algorithms studies that investigated the performance of DL algorithms for retinal vessel segmentation in digital fundus photographs. Our primary objective was to precisely gauge the performance of DL methods for retinal vessel segmentation from color fundus images. The evaluation of DL performance can help policymakers to understand how DL could be a clinically effective tool for segmenting retinal vessels in under-resourced areas with a severe shortage of experts and infrastructure.

## 2. Deep Learning (DL)

### 2.1. Artificial Neural Network (ANN)

ANNs are one of the main tools used in AI. *ANNs* are inspired by the neurons of a biological brain that is intended to mimic the way that humans learn. *ANN* consists of *input, hidden*, and *output* layers. The *input layer* is the first layer that receives inputs in the form of numbers, documents, texts, images, or audio files. The middle layer is called the *hidden layer*, and a single layer neural network is called a *perceptron*. However, it can consist of multiple layers and output single or multiple outcomes.

**Figure 1:**
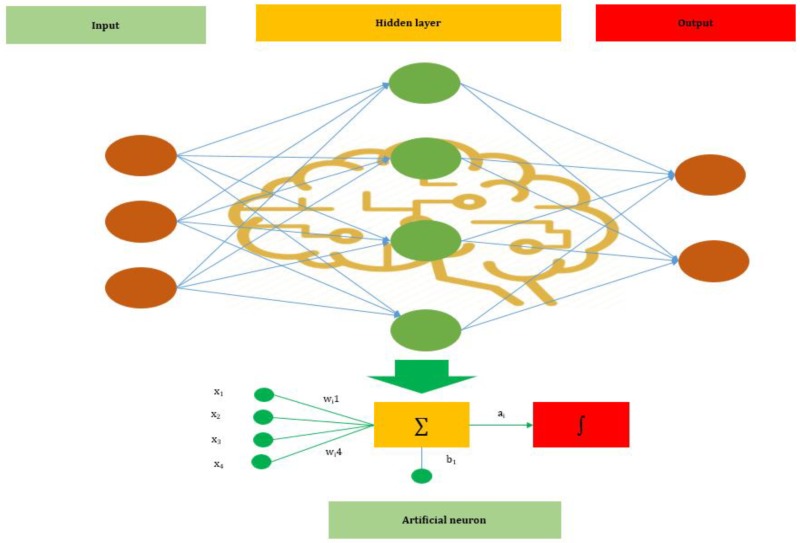
The basic structure of an Artificial Neural Network (ANN).

In Figure 1, $x_{1},$ $x_{2},$ $x_{3}$, and $x_{4}$ represent four inputs (independent variables) to the network. Each of the four inputs is multiplied by a random weight. The weights are represented as $w_{1}$, $w_{2}$, $w_{3}$, and $w_{4}$. Weight represents the strength of each node, while *b* is called the bias. A bias value lets the activation function go up or down. The following output is generated in the activation function:

(1) $$x_{1}.w_{1}+x_{2}.w_{2}+x_{3}.w_{3}+x_{4}.w_{4}$$

The activation function determines where a neuron would be activated or not by the sum of weights and with the addition of the bias. The primary objective is to introduce non-linearity into the output of each neuron.

### 2.2. Convolutional Neural Network (CNN)

**Figure 2:**
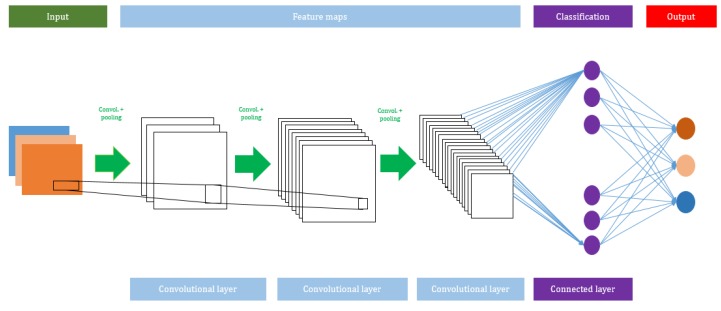
A schematic view of the Convolutional Neural Network (CNN) model.

A CNN algorithm consists of several network layers, such as *input*, *convolutional*, *max pooling, average pooling*, and *output layers*. The total number of layers can be increased or decreased based on the size of the input used to train the model. Usually, a deeper network will perform better with large datasets. The advantage of using a CNN is that it does not need any *feature extraction*. In the CNN model, the features are automatically hierarchically extracted from the input and they are further classified using a *fully connected layer*. Figure 2 shows the architecture of the CNN model.

#### 2.2.1. Convolutional Layer

The *convolutional layer* always utilizes a *convolutional function* on the given input variables, such as digital images. A filter is moved over the given input variables with a stride (which describes how many pixels a filter will be translating horizontally and vertically), and the size of the stride is usually determined by the providers. It generates feature maps and it is used as the input of the subsequent layer.

#### 2.2.2. Activation Function

Different types of *activation functions* are applied in the *convolutional layers*. They help to create a non-linear relationship between the data and the output class.

Let layer l be a non-linearity layer that takes the feature volume $Y_{I}^{\left(L-1\right)}$ from a convolutional layer (l−1) and generates the activation volume $Y_{i}^{\left(l\right)}$.

(2) $$Y_{i}^{\left(l\right)}=f(Y_{i}^{\left(l-1\right)})$$

There are several types of *activation functions*, such as tanh, sigmoid, or ReLu, which are used to classify output variables. However, ReLu is a widely used *activation function,* because of its capability to reduce the exploding/vanishing gradient problem.

Tan:f(x)=tanh(x)

$$\mathrm{Sigmoid}:f\left(x\right)=\frac{1}{1+e^{-x}}$$

ReLu:f(x)=max(0,x)

#### 2.2.3. Max Pooling

**Figure 3:**
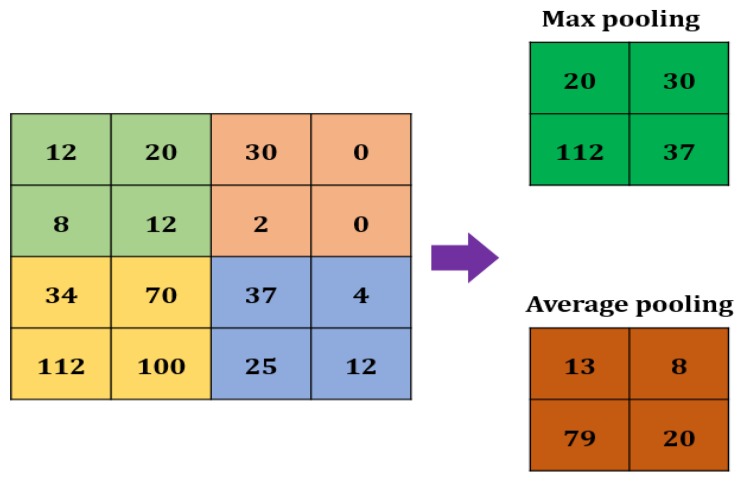
Max pooling in CNN.

A Max pooling layer is used to reduce the size of a feature. The value of the stride is selected according to the maximum value/average value (Figure 3). The maximum/average value is taken by the stride to generate a matrix. However, the output size of the layer is smaller than the previous layer.

#### 2.2.4. Fully Connected Layer

**Figure 4:**
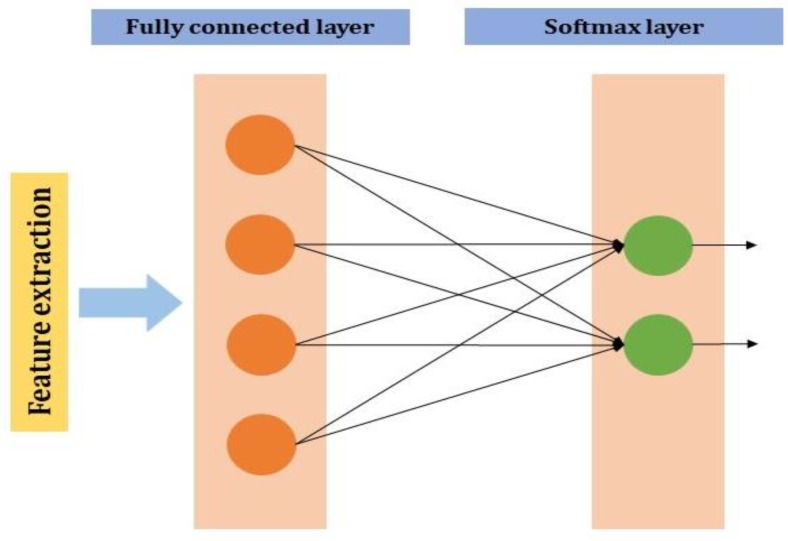
Fully connected layer in CNN.

The *neuron* of the previous layer i.e., the *max-pooling layer* is connected to each and every *neuron* in this layer. The output layer of the MLP will have $m_{1}^{\left(l-i\right)}$ outputs. In the output neurons, i denotes the number of the layer in the MLP (Figure 4).

If l−1 is a fully connected layer;

(3) $$y_{i}^{\left(l\right)}=f\left(z_{i}^{\left(l\right)}\right)\mathrm{with}z_{i}^{\left(l\right)}=\sum_{j=1}^{m_{1}^{\left(l-1\right)}}w_{i,j}^{\left(l\right)}y_{i}^{\left(l-1\right)}$$

### 2.3. Retinal Image Processing

The retinal vessel structure is compounded, and there are always immense differences between the vessels in various local areas in terms of size, shape, and intensity [14]. Thus, it is very difficult to build a model that can explain the compounded vessel structure. Some features are similar in shape and intensity with vessels (e.g., striped hemorrhage). Moreover, micro-vessels are very thin, and the width of the vessels varies (from one to several pixels), depending on the sizes and image resolutions. Therefore, it is challenging to differentiate retinal vessels from other similar features or noises. Multiple methods that were developed using vector geometry, image filters, and machine learning techniques have been used to generate the low-level feature vectors, which can detect the vessels [15,16]. The performance of these models sometimes relied on high-quality image features or heuristic presumptions. However, these traditional methods did not utilize generalized learning patterns to create feature vectors. Recently, deep learning algorithms have been used in retinal vessel segmentation, due to their ability to higher-level abstractions from diverse data by using multiple layers. Retinal vessel segmentation is conducted through pixel-wise processing. Vessel segmentation is considered to b a pixel-wise binary classification problem (vessel pixel versus non-vessel pixel). The CNN model with multiple layers differentiates images by analyzing them pixel by pixel, without considering the whole structure of the retinal vasculature [17]. The CNN model also combines multi-level features to provide higher segmentation performance. It can produce a vessel probability map while using the same size retinal images and a single forward propagation process.

## 3. Methods

### 3.1. Research Design

The Preferred Reporting Items for Systematic Reviews and Meta-Analyses (PRISMA), which is based on the Cochrane’s Handbook for Systematic Reviews, was used to conduct the current study [18,19]. A review of the written protocol was drafted (Supplementary Table S1). The process of this study is given below:

### 3.2. Search Methods for Identification of Studies

#### 3.2.1. Electronic Database Search

**Figure 5:**
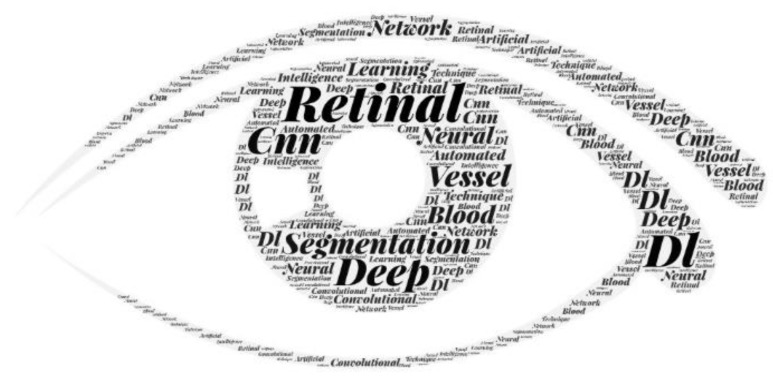
Search terms.

We systematically searched in the widely used search engines, namely EMBASE, PubMed, Google Scholar, Scopus, and Web of Science, to obtain potentially relevant studies that were published between 1 January 2000 and 15 January 2020, using the most appropriate free keywords (“Retinal vessel segmentation” or “Retinal blood segmentation” and (“Deep learning” or “DL” or “Convolutional neural network” or “CNN”, or “Deep neural network”, or “Automated technique”, or “Artificial intelligence”) (Figure 5).

#### 3.2.2. Searching for Other Sources

We also carefully searched the bibliography of obtained studies that we deemed to be eligible and relevant previous review studies for additional study inclusion.

### 3.3. Eligibility Criteria

Eligibility was restricted to studies that examined the performance of DL algorithms for retinal vessel segmentation while using digital images. Studies were included if they fulfilled the following inclusion criteria: (1) published in English, (2) provided an outcome of DL algorithms and retinal vessel segmentation, (3) provided information on any of the evaluation metrics, such as accuracy, the area under receiver operating curve, sensitivity, or specificity, (4) provided clear information about the image database and the number of images, (5) provided a clear definition of retinal vessel segmentation, and (6) clearly described the DL algorithms and process used in the retinal vessel segmentation.

Studies were excluded if they were published in the form of a review, editorial, research letter, letter to editor, or short communication.

### 3.4. Selection Process

Two authors (MMI, TNP) independently screened all of the titles and abstracts of previously obtained studies for inclusion in our systematic review and meta-analysis. They selected relevant studies that are based on the predefined selection criteria. Any disagreement at this stage was resolved by discussion with a prior agreement; any unsettled conflict was finally settled by discussion with the chief investigator (YC, L).

The same two authors used data collection forms to extract the relevant information from the previously obtained studies. MMI and TNP then assessed the obtained studies for duplication by comparing the publication date, author name, journal name, and sample sizes. Any duplicated study was excluded.

### 3.5. Data Extraction

The primary outcome measures were AUROC, sensitivity, and specificity of the performance of the DL algorithms for retinal vessel segmentation. We also recorded the total number of images used in the training and testing set. We also recorded data regarding the true positive, true negative, false positive, and false negative rate. Other data of interest included general information: author name, publication year, location, sensitivity, specificity, accuracy, AUROC, DL model, camera information, image pixels, and database.

### 3.6. Assessment of Bias Risk

Systematic reviews with meta-analysis of diagnostic studies might have heterogeneous findings due to differences in their study design [20]. Therefore, MMI and TNP independently utilized the Quality Assessment of Diagnostic Accuracy Studies-2 (QUADAS-2) tool for assessing the quality of the included diagnostic studies. The QUADAS-2 scale [21] comprises four domains: patient selection, index test, reference standard, and flow and timing. The first three domains are used for evaluating the risk of bias in terms of concerns regarding applicability. The overall risk of bias was categorized into three groups (low, high, and unclear risk bias) (Supplementary Table S2).

### 3.7. Statistical Analysis

The Meta-Disc (Version: 1.4, U. de Bioestadística, Madrid (España)) software was used to calculate the evaluation metrics, such as AUROC, sensitivity, specificity, and diagnostic odds ratio. The Meta-Disc was used to (a) perform statistical pooling from each individual study, (b) assess the homogeneity with a variety of statistics, including chi-square and I-squared. Six evaluation criteria were developed, including the area under the ROC (AUROC) curve, sensitivity (SN), specificity (SP), positive likelihood ratio (LR+), negative likelihood ratio (LR−), and diagnostic odds ratio.

The value of the AUROC curve ≥90, <0.90, <0.80, <0.70, and <0.60 were considered to be excellent, good, fair, poor, and failed, respectively. An *I*^2^ value was calculated to assess the statistical heterogeneity among the included studies. An *I*^2^ value of 0∼25%, 25∼50%, 50∼75%, and >75% were considered as very low, low, medium, and high heterogeneity, respectively [7]. The value of *I*^2^ was computed, as follows:

(4) $$I^{2}=\frac{100\%\times \left(Q-d.f\right)}{Q}$$

Here, Q = Cochrane’s heterogeneity statistic and df = degree of freedom. Negative values of $I^{2}$ are considered as zero; the $I^{2}$ value is between 0% (no observed heterogeneity) and 100% (maximum heterogeneity). It allows for calculating the AUC and Q* index, along with their standard errors.

The *SE*, *SP*, *LR*+, *LR*−, and diagnostic odds ratio are defined, as follows:

(5) $$SE=\frac{TP}{TP+FN}$$

(6) $$SP=\frac{TN}{TN+FP}$$

where *TP* = Vessel pixels classified correctly, *FN* = Vessel pixels misclassified as non-vessel pixels, *TN* = Non-vessel pixels classified correctly, and *FP* = Non-vessel pixels misclassified as vessel pixels. The diagnostic odds ratio (DOR) was also computed for assessing how much greater the odds of having DR are for the people with a positive test result than for the people with a negative test result. DOR is calculated by the following equation,

(7) $$DOR=\frac{Positive\;likelihood\;ratio\left(LR+\right)}{Negative\;likelihood\;ratio\left(LR-\right)}=\frac{TP.TN}{FN.FP}.$$

The likelihood ratios were calculated to express how much more frequent the respective finding is among the individuals with DR than among the individuals without DR.

(8) $$LR+=\frac{SE}{1-SP}$$

(9) $$LR-=\frac{\left(1-SE\right)}{SP}$$

The pooled AUROC was plotted with the *SE* versus (1 − *SP*) by varying the threshold. The perfect classifier achieved an AUC value that was equal to 1.

## 4. Results

### 4.1. Study Screening

**Figure 6:**
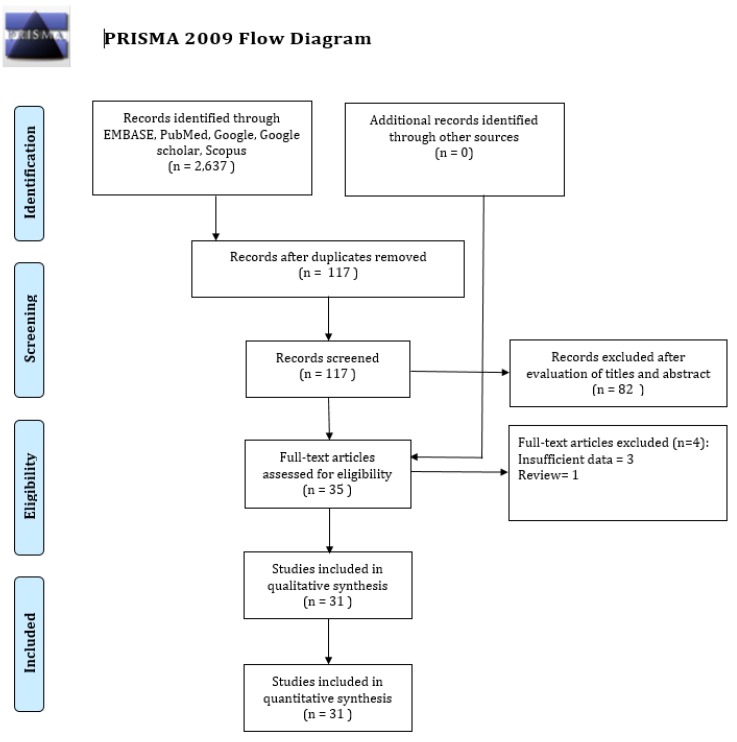
PRISMA (Preferred Reporting Items for Systematic Reviews and Meta-Analyses) flow diagram for study selection.

Our initial studies search of the five search engines yielded 2637 studies. 2520 studies were excluded because of duplication, and 82 out of 117 studies were excluded after reviewing the titles and abstracts that were based on our pre-specified inclusion criteria. We then reviewed the remaining 35 full-text studies and checked their reference lists for further relevant studies. However, we did not find any additional potential study. Three more studies were excluded for insufficient data, and one study was excluded because it was a review. Consequently, we included the remaining 31 studies for this systematic review and meta-analysis [11,12,13,22,23,24,25,26,27,28,29,30,31,32,33,34,35,36,37,38,39,40,41,42,43,44,45,46,47,48,49]. Figure 6 presents the flow diagram of the systematic studies search.

### 4.2. Study Characteristics

**Figure 7:**
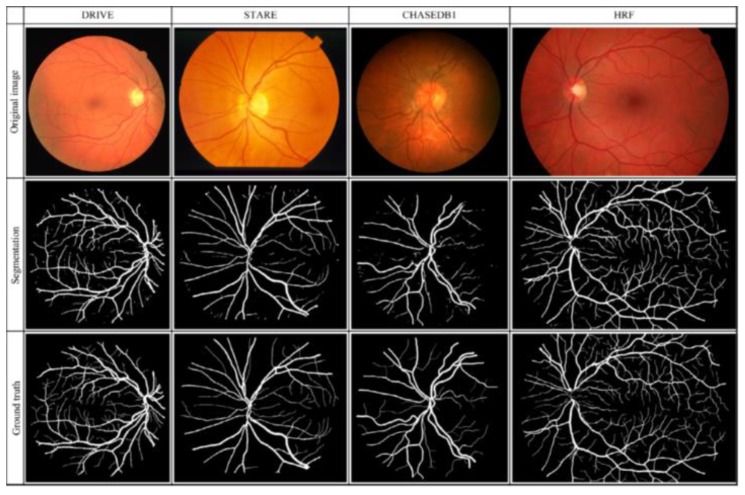
Original retinal, segmentation, and ground truth images in the four different databases.

Table 1 presents a total of 31 studies that evaluated the performance of DL algorithms for retinal vessel segmentation. The publication years ranged from 2015 to 2019. All of the studies used DL algorithms, like CNN, MResU-Net, or U-Net, for retinal vessel segmentation. The range of accuracy and AUROC was between 0.85 and 0.99. Seven types of publicly available databases, such as DRIVE, STARE, CHASEDB1, HRF, TONGREN, DRIONS, and REVIEW, were used in their studies (Table 2). The REVIEW database only had 16 images and the DRIONS database had a maximum number of 110 images. Each image had pixel-level vessel annotation provided by experts, and ground truth was used for image annotation (Figure 7).

### 4.3. Deep Learning Performance in Retinal Vessel Segmentation

**Figure 8:**
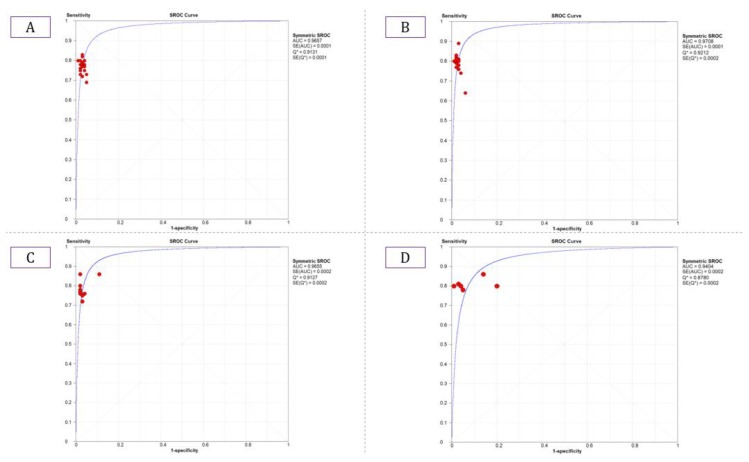
Summarized ROC curve of deep learning (DL) algorithms (**A**) DRIVE, (**B**) STARE, (**C**) CHASE_DB1, and (**D**) HRF.

The summary estimate for the retinal vessel segmentation sensitivity of DL systems was 0.77 (95% CI: 0.77–0.77) and the specificity was 0.97 (95%CI, 0.97–0.97) based on the 23 studies that utilized the DRIVE data set (Table 3). The summarized AUROC was 0.96 (Figure 8).

**Figure 9:**
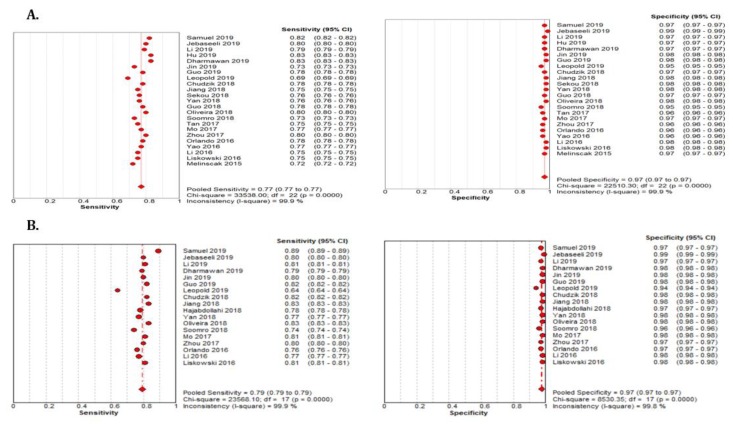
Performance of the DL model retinal vessel segmentation (**A**) pooled sensitivity and specificity of DRIVE dataset (**B**) pooled sensitivity and specificity of the STARE dataset.

**Figure 10:**
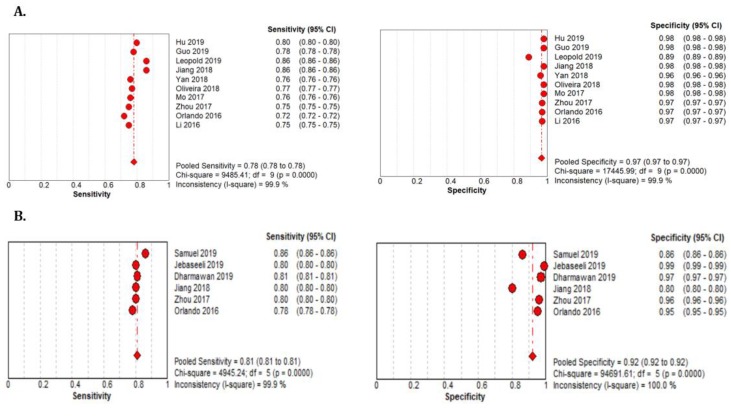
Performance of the DL model retinal vessel segmentation (**A**) pooled sensitivity and specificity of CHASEDB1 dataset (**B**) pooled sensitivity and specificity of the HRF dataset.

The 18 studies that used the STARE data sets had significantly higher sensitivity but the same specificity as the DRIVE data set. The summarized AUROC was 0.97, and the pooled sensitivity and specificity were 0.79 (95% CI: 0.79–0.79) and 0.97 (95%CI, 0.97–0.97), respectively (Figure 9). The 10 studies that used the CHASEDB1 data set for evaluating the performance of DL in retinal vessel segmentation had a summarized AUROC of 0.96, and the pooled sensitivity and specificity were 0.78 (95%CI, 0.78–0.78) and 0.97 (95%CI, 0.97–0.97), respectively (Figure 10). Furthermore, six studies that used the HRF data set to assess the performance of DL in retinal vessel segmentation had a summarized AUROC of 0.94, and the pooled sensitivity and specificity were 0.81 (95%CI, 0.81–0.81) and 0.92 (95%CI, 0.92–0.92) (Table 3), respectively (Supplementary Figure F1-F4).

### 4.4. Performance Comparison for Models in the Different Databases

Table 4 provides the performance of the unsupervised models that were proposed in the literature in relations of the typical incoherency measurements.

## 5. Discussion

### 5.1. Principal Findings

This systematic review with meta-analysis assessed the performance of the automated DL algorithms for retinal vessel segmentation from fundus retinal images. Our key findings are: (a) DL algorithms showed great performance when they assessed images that were available from four publicly available databases in terms of sensitivity and specificity; and, (b) the performance of DL was comparable to that of human experts (Table 3). Our findings suggest that the application of DL-based tools for retinal vessel segmentation could provide a substitute solution for eye screening, especially in clinical settings with a limited number of ophthalmologists and a scarcity of resources. The implementation of AI screening tools in real-world clinical settings can speed up the screening process, reduce cost, and improve patients care, since the performances of DL-based tools and human graders were similar.

### 5.2. Research and Clinical Implications

Automatic segmentation of retinal vessels is one of the most important elements in precision treatment dealing with huge datasets of retinal images. Manual segmentation is time-consuming and complex due to its structure and variability across human graders [41,53]. The automatic tool is clinically effective in segmenting retinal images, and identification might improve accurate diagnosis by non-retinal experts; therefore, the application of automatic tools to analysis of retinal images could provide an alternative solution for large-scale fundus images screening, especially in areas with limited access to ophthalmologic experts [6]. However, the automatic segmentation of retinal images is not an easy task, and several factors, including light exposure, camera focus, motion artifact, and existing diseases, can hamper the image quality [54,55,56]. These potential factors are often responsible for inhomogeneous image quality and, thus, hamper vessel segmentation. Accordingly, extensive efforts have been made to segment retinal vessels automatically while using machine learning techniques, but failed to show superior performance over human graders.

The DL algorithms have shown promising performance comparable to expert segmentation in fundus images [28,38]. The most unique advantage of DL is the ability to precisely learn and capture a huge amount of image features with varying hierarchies and locations. It has the great ability to optimally integrate these features to obtain a desirable finding. The results of our study show that the DL algorithms were able to segment retinal vessels with performances that were comparable to that of human experts. The accurate segmentation of retinal vessels assists in making appropriate clinical decisions. It will help for screening high-risk patients that need to receive proper treatment, such as retinopathy, whereas accurate segmentation can guide retinal disease management. DL-based automatic tools in retinal vessel segmentation could markedly change how retinal disease diagnosis and management is conducted in the near future. Automatic segmentation of retinal vessels could become popular in an era where fast, accurate, and low-cost treatment is recommended [40]. It would be particularly helpful for ophthalmologists who are not trained experts in retinal image identification. It would also assist experienced eye specialists to make a decision quicker and more accurately. Precise risk stratification for eye disease treatment, such as glaucoma, DR, would become possible. However, high quality of the image database is a prerequisite for successfully implementing DL-based automatic tools into retinal vessel segmentation.

### 5.3. Strengths and Limitations

Our study has several strengths that need to be addressed. First, this is the first systematic review and meta-analysis that addressed DL performance in retinal vessels detection. Second, this study included a total of 31 studies that had used seven different databases to assess the performance of DL. Our results indicate that DL has immense potential to improve care. Third, we have compared the DL performance with the performance of human experts and other types of unsupervised methods. Our study also has some limitations. First, more than two-thirds of the included studies used the same three databases (namely, DRIVE, STARE, HRF); therefore, we cannot generalize our results as much as if more databases had been involved in our meta-analysis. However, some studies used the remaining four databases and achieved similar performances. Second, we did not include any study that evaluated a machine learning model to assess retinal vessels detection. Third, inherited retinal degeneration diseases are genetically heterogeneous. Therefore, changes in retinal vessels in fundus images could be different between patients with the same retinal diseases, and the performance of deep learning could vary. However, a robust design and a trained CNN model or novel post-processing can solve this problem.

## 6. Conclusions

Our current study findings show that DL algorithms achieved clinically acceptable performances in retinal vessel segmentation. The implementation of DL-based tools in retinal vessel segmentation can reduce manpower, the cost of retinal vessel screening, and resolve the problem of intra-grader and inter-grader variability. In the near future, DL techniques may play a significant role in determining ophthalmological diseases and predicting the prognosis for eye disease patients in an individualized manner. More careful, comprehensive designs and planning are needed in order to expedite this process.

## Figures and Tables

**Table 1:** Characteristic of included studies.

Author	Year	Model	Dataset	SN/SP	Accuracy	AUROC
Samuel [22]	2019	CNN	DRIVE	0.82/0.97	0.96	0.98
			STARE	0.89/0.97	0.96	0.99
			HRF	0.86/0.86	0.85	0.96
Jebaseeli [23]	2019	TPCNN	DRIVE	0.80/0.99	0.98	-
			STARE	0.80/0.99	0.99	-
			REVIEW	0.80/0.98	0.99	-
			HRF	0.80/0.99	0.98	-
			DRIONS	0.80/0.99	0.99	-
Li [24]	2019	MResU-Net	DRIVE	0.79/0.97	-	0.97
			STARE	0.81/0.97	-	0.98
Hu [25]	2019	S-UNet	DRIVE	0.83/0.97	0.95	0.98
			CHASEDB1	0.80/0.98	0.96	0.98
			TONGREN	0.78/0.98	0.96	0.98
Dharmawan [26]	2019	Hybrid U-Net	DRIVE	0.83/0.97	-	0.97
			STARE	0.79/0.98	-	0.98
			HRF	0.81/0.97	-	0.98
Jin [27]	2019	CNN	DRIVE	0.73/0.98	0.96	0.97
			STARE	0.80/0.98	0.96	0.98
Guo [28]	2019	BTS-DSN	DRIVE	0.78/0.98	0.95	0.98
			STARE	0.82/0.98	0.96	0.98
			CHASEDB1	0.78/0.98	0.96	0.98
Leopold [29]	2019	PixelBNN	DRIVE	0.69/0.95	0.91	0.82
			STARE	0.64/0.94	0.90	0.79
			CHASEDB1	0.86/0.89	0.89	0.87
Lin [30]	2018	CNN	DRIVE	0.76/-	0.95	-
			STARE	0.74/-	0.96	-
			CHASEDB1	0.78/-	0.95	-
Chudzik	2018	CNN	DRIVE	0.78/0.97	-	0.96
			STARE	0.82/0.98	-	0.98
Jiang [31]	2018	CNN	DRIVE	0.75/0.98	0.96	0.98
			STARE	0.83/0.98	0.97	0.99
			CHASEDB1	0.86/0.98	0.96	0.98
			HRF	0.80/0.80	0.96	0.97
Sekou [32]	2018	CNN	DRIVE	0.76/0.98	0.95	0.98
Hajabdollahi [33]	2018	CNN	STARE	0.78/0.97	0.96	-
Yan [34]	2018	CNN	DRIVE	0.76/0.98	0.95	0.97
			STARE	0.77/0.98	0.96	0.98
			CHASEDB1	0.76/0.96	0.94	0.96
Guo [35]	2018	MDCNN	DRIVE	0.78/0.97	0.95	0.97
			STARE	-	-	-
Oliveira [36]	2018	CNN	DRIVE	0.80/0.98	0.95	0.98
			STARE	0.83/0.98	0.96	0.99
			CHASEDB1	0.77/0.98	0.96	0.98
Soomro [37]	2018	CNN	DRIVE	0.73/0.95	0.94	0.84
			STARE	0.74/0.96	0.94	0.85
Tan [38]	2017	CNN	DRIVE	0.75/0.96	-	-
Mo [39]	2017	CNN	DRIVE	0.77/0.97	0.95	0.97
			STARE	0.81/0.98	0.96	0.98
			CHASEDB1	0.76/0.98	0.95	0.98
Zhou [40]	2017	CNN	DRIVE	0.80/0.96	0.94	-
			STARE	0.80/0.97	0.95	-
			CHASEDB1	0.75/0.97	0.95	-
			HRF	0.80/0.96	0.95	-
Dasgupta [13]	2017	CNN	DRIVE	-	0.95	0.97
Şengür [41]	2017	CNN	DRIVE	-	0.91	0.96
Orlando [42]	2016	CNN	DRIVE	0.78/0.96		0.95
			STARE	0.76/0.97		-
			CHASEDB1	0.72/0.97		0.95
			HRF	0.78/0.95		0.93
Yao [43]	2016	CNN	DRIVE	0.77/0.96	0.93	-
Li [44]	2016	CNN	DRIVE	0.75/0.98	0.95	0.97
			STARE	0.77/0.98	0.96	0.98
			CHASEDB1	0.75/0.97	0.95	0.97
Maji [45]	2016	CNN	DRIVE	-	0.94	-
Lahiri [46]	2016	CNN	DRIVE	-	0.95	0.95
Liskowski [47]	2016	CNN	DRIVE	0.75/0.98	0.95	0.97
			STARE	0.81/0.98	0.96	0.98
Fu [48]	2016	CNN	DRIVE	0.72/-	0.94	-
			STARE	0.71/-	0.95	-
Fu [11]	2016	CNN + CRF layer	DRIVE	0.76/-	0.95	-
			STARE	0.74/-	0.95	-
			CHASEDB1	0.71/-	0.94	-
Melinscak [12]	2015	CNN	DRIVE	0.72/0.97	0.94	0.97

**Table 2:** Description of databases.

Dataset	Number of Image	Description	Camera	Resolution (Pixel)	Dataset Partition
DRIVE	40	Dataset was collected from 400 diabetic patients aged between 25 and 90 years. 40 photographs were randomly selected, 33 did not show any sign of DR, and 7 showed signs of mild early DR. Training set: Single manual segmentation Testing set: Two manual segmentation	Canon CR5 nonmydriatic 3CCD camera with a 45° field of view (FOV)	565 × 584	Yes Training: 20 Testing: 20
STARE	20	Images were collected from DR, PDR, ASR, HTR, etc. patients. Each image has pixel-level vessel annotation provided by two experts. Performance is computed with the segmentation of the first observer as ground truth.	TopCon TRV-50 fundus camera with a 35° FOV	700 × 605	No
CHASE_DB1	28	Subset of retinal images of multiethnic children from the Child Heart and Healthy Study in England. (https://blogs.kingston.ac.uk/retinal/chasedb1/)	Nidek NM-200-D fundus camera with a 30° FOV	1280 × 960	Yes Training: 20 Testing: 8
HRF	45	Data were collected from 15 healthy patients, 15 glaucomatous patients, and 15 diabetic retinopathy patients separately. It contains a binary gold standard vessel segmentation images that are determined by a group of experts (experience in retinal images analysis).	Canon CR-1 fundus camera with a field of view of 45° and different acquisition setting	500 × 2500	No
TONGRE	30	Images collected from 30 people at the Tongren Beijing Hospital, where five of these images show a pathological pattern (glaucoma).	NR	1880 × 281	Yes Training: 15 Test: 15
DRIONS	110	Dataset contains high resolution images of blood vessels, 25 images were from patients with chronic glaucoma while the remaining 85 images were from hypertensive retinopathy patients.	Analogical fundus camera approximately centered on the ONH	600 × 400	Yes Training: 60 Test: 50
REVIEW	16	The dataset includes retinal images with 193 vessel segments, demonstrating a variety of pathologies, and vessel types (8 high-resolution, 4 vascular diseases, 2 central light reflex, 2 kickpoint). It also contains 5066 manually marked profiles. It has been marked by three observers.	NR	1360 × 1024 to 3584 × 2438	No

DRIVE = The Digital Retinal Images for Vessel Extraction database; STARE = The Structured Analysis of the Retina database; CHASE_DB1 = The Child Heart and Health Study in England database; HRF = High-Resolution Fundus; REVIEW = Retinal Vessel Image set for Estimation of Widths, DR = Diabetic retinopathy, PDR = Proliferative diabetic retinopathy, ASR = Arteriosclerotic Retinopathy, HTR = Hypertensive Retinopathy.

**Table 3:** Summary Estimates of DL performance in retinal vessel segmentation.

	SE with 95% CI	SP with 95% CI	LR+ with 95% CI	LR− with 95% CI	DOR with 95% CI
**DRIVE**					
Human experts	0.77	0.97	NR	NR	NR
DL *	0.77 (0.77–0.77)	0.97 (0.97–0.97)	28.19 (24.21–32.82)	0.23 (0.22–0.25)	120.57 (99.66–145.86)
**STARE**					
Human experts	0.89	0.93	NR	NR	NR
DL *	0.79 (0.79–0.79)	0.97 (0.97–0.97)	31.02 (30.77–31.28)	0.21 (0.21–0.21)	136.67 (135.42–137.0)
**CHASE_DB1**					
Human experts	0.83	0.97	NR	NR	NR
DL *	0.78 (0.78–0.78)	0.97 (0.97–0.97)	22.97 (22.75–23.20)	0.23 (0.23–0.23)	109.27 (108.0–110.56)
**HRF**					
Human experts	NR	NR	NR	NR	NR
DL *	0.81 (0.81–0.81)	0.92 (0.92–0.92)	10.32 (10.26–10.38)	0.21 (0.21–0.21)	51.75 (51.35–52.16)

* Note: DL = Deep Learning, NR = Not Reported, SE = Sensitivity, SP = Specificity, LR = Likelihood Ratio, CI = Confidence Interval, * = Summarized.

**Table 4:** Performance comparison with unsupervised methods for retinal vessel segmentation.

Methods	SN	SP	ACC	AUC
**DRIVE**				
**Unsupervised**				
Azzopardi et al. [50]	0.76	0.97	0.94	0.96
Zhang et al. [51]	0.77	0.97	0.94	0.96
Roychowdhury et al. [52]	0.73	0.97	0.94	0.96
**STARE**				
**Unsupervised**				
Azzopardi et al. [50]	0.77	0.97	0.94	0.95
Zhang et al. [51]	0.77	0.97	0.95	0.97
Roychowdhury et al. [52]	0.73	0.98	0.95	0.96
**CHASE_DB1**				
**Unsupervised**				
Azzopardi et al. [50]	0.75	0.95	0.93	0.94
Zhang et al. [51]	0.76	0.96	0.94	0.96
Roychowdhury et al. [52]	0.76	0.95	0.94	9.96

Note: SE = Sensitivity, SP = Specificity, ACC = Accuracy.

## Supplementary Materials

The following are available online at https://www.mdpi.com/2077-0383/9/4/1018/s1. Table S1: PRISMA 2009 Checklist; Table S2: Quality Assessment of Diagnostic Accuracy Studies-2 for Included Studies; Supplementary Figure F1-F4: Performance of DL in Retinal Vessel Segmentation:
